# A real-world data study on long-course venetoclax combined with hypomethylating agents in unfit patients

**DOI:** 10.3389/fonc.2026.1826350

**Published:** 2026-04-21

**Authors:** Chen Cao, Minran Zhou, Xinwen Jiang, Qingli Ji, Xiaoqing Li, Sai Ma, Chunyan Chen

**Affiliations:** 1 Department of Hematology, Qilu Hospital, Shandong University, Jinan, China; 2 Shandong Key Laboratory of Hematological Diseases and Immune Microenvironment, Jinan, China; 3 Shandong Provincial Clinical Research Center for Hematological Diseases, Jinan, China

**Keywords:** acute myeloid leukemia, hypomethylating agents, long-course treatment, measurable residual disease, unfit patients, venetoclax

## Abstract

This study aimed to investigate the efficacy, safety and prognostic value of measurable residual disease (MRD) in unfit AML patients treated with long-course venetoclax combined with hypomethylating agents, so as to provide evidence for optimizing clinical low-intensity treatment regimens.A single-center retrospective analysis was conducted on unfit AML patients receiving this regimen. Clinical data were collected to explore the correlation between dynamic MRD changes at different treatment stages and patient prognosis.The long-course regimen exhibited stable clinical efficacy and effectively achieved disease remission. MRD negativity was closely associated with better prognosis, while patients with MDS-transformed AML and relapsed/refractory AML had higher recurrence risk. Adverse reactions were mainly hematological and infectious non-hematological toxicity, with controllable overall safety.Dynamic MRD monitoring provides valuable prognostic information in unfit AML patients receiving venetoclax plus hypomethylating agents. Sustained MRD negativity predicted favorable outcomes, highlighting the clinical utility of MRD-guided strategies. Due to the absence of a short-course control group, causal conclusions about treatment duration cannot be drawn, and survivorship bias should be considered. Study limitations include single-center design and limited sample size; conclusions require validation by larger multicenter prospective studies.

## 1 Introduction

Acute myeloid leukemia (AML) can occur at any age, but it is primarily a disease of older adults, with a median age at diagnosis of 68 years (1). The majority of patients still have no identifiable predisposing factors for AML. A prior history of hematological disorders, including myelodysplastic syndromes or myeloproliferative neoplasms, is also associated with a significantly increased risk of progression to AML (2, 3). Before the advent of venetoclax, outcomes for older patients with AML or those unfit for intensive chemotherapy were generally poor. For patients aged 70 years and older, a European multicenter study analyzing 1,199 patients who received intensive chemotherapy reported a composite complete remission rate of 56% and a median overall survival (OS) of 6.85 months (95% CI 3.7–13.5 months) for all patients. The early (30-day) mortality rate was 11%, highlighting an urgent need for more effective and less toxic treatment options (4). AML cells express B-cell lymphoma-2 (BCL-2), enabling them to bind pro-apoptotic proteins and evade apoptosis. Venetoclax is a potent BCL-2 inhibitor specifically targeting BCL-2. Following the publication of the pivotal randomized controlled trial VIALE-A, venetoclax in combination with azacitidine (VEN+AZA) became the standard of care for AML patients aged 75 years or older or those unfit for intensive chemotherapy (5). As first-line therapy for newly diagnosed, untreated AML patients, VEN+AZA increases complete remission rates and improves median overall survival compared to azacitidine alone (OS: 14.7 vs. 9.6 months; hazard ratio for death, 0.66; 95% confidence interval, 0.52 to 0.85; P<0.001) (6). Furthermore, VEN combined with AZA is also used in the treatment of patients with relapsed/refractory AML and has demonstrated favorable efficacy and improved survival in clinical practice. Several studies have further shown that the use of venetoclax combined with HMAs results in a 43%∼46% ORR in patients with relapsed/refractory acute myeloid leukemia (7, 8).

Despite the significant progress achieved with venetoclax combined with hypomethylating agents (HMA) in the treatment of AML, data regarding the efficacy, safety, and long-term prognosis of extended courses (≥4 cycles) of VEN+HMA regimens remain scarce. Existing evidence primarily focuses on the outcomes of short-course therapy, whereas the impact of long-term maintenance treatment on sustained disease control, reduction of relapse risk, and prolongation of survival has not been fully elucidated. To address this gap, this study conducted a retrospective analysis based on single-center clinical data from unfit AML patients who received extended courses of VEN+HMA therapy, aiming to evaluate the real-world efficacy and safety of this treatment strategy and to provide important insights for clinical decision-making.

## 2 Materials and methods

### 2.1 Patient cohorts

This is a single-center retrospective study of unfit acute myeloid leukemia patients who received extended courses of VEN+HMA therapy at Qilu Hospital of Shandong University between December 2018 and December 2025. Key inclusion criteria were age ≥18 years, a confirmed diagnosis of AML according to World Health Organization criteria, and receipt of at least four cycles of VEN-HMA treatment. Patients with acute promyelocytic leukemia or uncontrolled infections were excluded from this study. Among the 79 enrolled patients, three patients had acute myeloid leukemia secondary to myelodysplastic syndromes (MDS/AML), two patients had relapsed/refractory acute myeloid leukemia (R/R AML), and the remaining 74 were newly diagnosed patients with acute myeloid leukemia who were unfit for intensive chemotherapy.

### 2.2 Methods

Statistical analysis was performed using SPSS software (version 27.0). Categorical data were presented as counts or rates. For measurement data, variables conforming to a normal distribution were expressed as mean ± standard deviation (SD), while those not conforming to a normal distribution were expressed as median (interquartile range). Median overall survival (OS) and progression-free survival (PFS) were estimated using the Kaplan-Meier method.

### 2.3 Definitions

ELN 2024 genetic risk classification for low-intensity therapy was defined according to the European LeukemiaNet 2024 recommendations:

Low-risk: patients with NPM1 mutation (without FLT3-ITD, NRAS, KRAS, or TP53 mutations); IDH2 mutation (without FLT3-ITD, NRAS, KRAS, or TP53 mutations); IDH1 mutation (without TP53 mutation); DDX41 mutation; or other genetic abnormalities (without FLT3-ITD, NRAS, KRAS, or TP53 mutations).

Intermediate-risk: patients with other genetic abnormalities accompanied by FLT3-ITD and/or NRAS mutation and/or KRAS mutation (without TP53 mutation).

High-risk: patients with TP53 mutation.

Complete remission (CR) required <5% marrow blasts, absence of extramedullary disease, and count recovery; CR with incomplete hematologic recovery (CRi) required all CR criteria except for persistent cytopenias. Relapse was defined as the reappearance of blasts in the peripheral blood, bone marrow (≥5%), or extramedullary sites after achieving CR. Measurable residual disease (MRD) negativity was defined as <0.1% leukemic cells detected by flow cytometry post-induction. Overall survival (OS) was calculated from the date of diagnosis to death from any cause or last follow-up. Progression-free survival (PFS) was defined as the time from treatment initiation to disease progression or death from any cause. Adverse events were graded by Common Terminology Criteria for Adverse Events (CTCAE) v5.0.

## 3 Results

### 3.1 Patient characteristics

A total of 79 patients were included in this study (**Table 1**). All 79 patients had available genetic sequencing results for myeloid neoplasms (**Table 2**). The cohort comprised 42 males (53.1%) and 37 females (46.9%), with a median age of 61 years (range, 33–82 years); patients aged >60 years accounted for 60.7%, and those with an ECOG performance status ≥2 accounted for 15.1%. The median white blood cell count was 3.93 (range, 0.24-167.57) × 10^9^/L, hemoglobin was 75 (range, 48-143) g/L, and platelet count was 41 (range, 4-429) × 10^9^/L. The median bone marrow blast percentage was 64.5% (range, 8-94%). FAB M5 subtype accounted for 62% (49/79) of cases. Among patients with available karyotype results, abnormal karyotype was observed in 39.39% (26/66), and adverse-risk karyotype in 12.6% (10/66). According to the ELN 2024 genetic risk classification for low-intensity therapy, 63.2% (50/79) of patients were classified into the low-risk group, 30.3% (24/79) into the intermediate-risk group, and 6.3% (5/79) into the high-risk group. The median number of cycles of venetoclax combined with hypomethylating agents was 6 (28 days per cycle). The dose of venetoclax was 400 mg daily (200 mg daily if patients were receiving concurrent azole antifungals). Prior treatment with HMA was not an exclusion criterion.

**Table 1:** Baseline clinical and genetic features of the study population.

Patient	Gender	Age(years)	WBC 10^9^/L	HGB g/L	PLT 10^9^/L	BM(%)	FAB
1	male	33	2.14	96	62	80%	/
2	female	59	2.73	54.0	53	47%	/
3	male	68	1.36	97	123	43%	M5
4	male	71	1.91	77.0	25	21%	M5
5	male	59	1.93	53	82	27%	/
6	male	60	9.87	87.0	22	68%	M2
7	female	75	1.49	68.0	31	64%	M2
8	male	70	0.79	65.0	18	8%	M4
9	male	73	1.15	80.0	26	65%	/
10	male	61	1.08	105.0	61	57%	/
11	male	64	2.18	143.0	179	23%	M5
12	male	74	8.34	114	31	32%	M5
13	male	51	1.41	52.0	123	84%	/
14	female	35	1.92	68.0	21	88%	/
15	male	63	10.13	82.0	20	45%	M5
16	male	75	3.37	83.0	14	27%	M5
17	female	69	5.17	122	35	/	/
18	male	57	3.16	55	37	75%	M2
19	female	59	29.04	103	98	82%	M5
20	female	55	1.08	66.0	11	86%	M5
21	female	52	3.30	68.0	32	68%	M5
22	female	38	1.36	65.0	78	80%	/
23	male	51	30.28	65.0	73	67%	M5
24	male	71	2.43	75.0	41	20%	/
25	male	61	10.53	68	22	75%	M5
26	female	68	21.31	67.0	170	31%	M5
27	female	72	0.86	53.0	34	68%	/
28	female	59	0.33	67	69	94%	M5
29	female	34	68.38	51	47	75%	M5
30	female	55	3.24	52	49	86%	M2
31	female	49	4.32	66.0	107	71%	M5
32	female	60	7.75	75	4	87%	M5
33	male	72	0.24	70.0	35	70%	M2
34	male	51	5.63	84.0	39	64%	M5
35	female	74	100.27	109.0	37	90%	M5
36	male	66	5.71	138.0	17	23%	M5
37	female	64	4.41	65.0	38	38%	/
38	male	34	3.93	131	4	70%	M5
39	female	62	2.05	78.0	251	65%	M5
40	female	53	5.68	76.0	197	32%	M5
41	male	64	36.54	116	29	89%	M5
42	male	63	167.57	57.0	21	94%	M5
43	male	56	27.19	68.0	27	64%	M5
44	female	69	1.82	81.0	56	63%	M5
45	male	70	15.85	94.0	93	59%	M5
46	male	69	41.57	78.0	31	80%	M2
47	female	53	15.68	56.0	30	85%	M5
48	female	33	5.12	82.0	100	69%	M5
49	female	57	44.02	60.0	34	80%	M5
50	male	72	16.09	83.0	31	26%	M5
51	male	47	1.77	109.0	144	29%	M5
52	female	82	2.05	74	35	22%	M5
53	female	78	6.95	75.0	163	80%	M5
54	female	59	1.15	75.0	30	84%	/
55	male	68	48.72	115.0	43	81%	M5
56	male	69	3.09	96.0	402	26%	M2
57	male	54	45.19	73.0	28	44%	M4
58	male	55	2.56	69.0	37	30%	M4
59	female	56	16.00	89.0	215	55%	/
60	female	72	36.98	62.0	122	82%	M5
61	male	60	7.16	95.0	43	53%	M5
62	female	74	2.44	102.0	249	61%	M5
63	female	53	64.27	91.0	429	92%	M5
64	male	67	1.90	69.0	69	40%	M5
65	female	70	2.92	68.0	139	90%	M4
66	female	53	9.89	55.0	103	34%	/
67	male	60	2.49	63.0	14	12%	M5
68	male	60	8.95	107.0	32	32%	M5
69	female	65	0.92	81.0	128	71%	M1
70	male	64	0.36	70.0	51	23%	/
71	male	37	4.56	118.0	20	30%	M5
72	female	74	1.41	88.0	80	22%	/
73	male	61	21.59	96	17	30%	M5
74	female	67	4.95	93.0	51	65%	M5
75	male	60	1.69	106.0	38	58%	M5
76	male	65	14.83	74.0	65	71%	M5
77	female	60	131.73	63.0	67	91%	M5
78	female	65	1.80	80.0	162	73%	M5
79	male	57	3.12	48.0	40	52%	/

WBC white blood cell count, HGB hemoglobin, PLT platelet count, BM bone marrow, FAB French-American-British classification.

**Table 2:** Peripheral blood and bone marrow parameters of the study population.

Clinical characteristics	Total *N* = 79
age, median (min, max)	61(33–82)
no. (%)	31(39.30%)
≥60y, no. (%)	48(60.70%)
Sex, no. (%)	
Male	42(53.10%)
Female ECOG PS 0-1 ≥2	37(46.90%) 67(84.9%) 12(15.1%)
FAB subtype, no. (%)	
M5	49(62.00%)
other	40(38.00%)
WBC, median (IQR)	3.93(0.24–167.57)
Hb, median (IQR)	75(48-143)
PLT, median (IQR)	41(4-429)
Bone marrow blast count (%), median (IQR)	64.5(8–94)
ELN risk group, no. (%)	
Favorable	50 (63.30%)
Intermediate	24 (30.40%)
Adverse	5 (6.30%)
Cytogenetic risk category, no. (%)	
Normal	39/65(60.00%)
7 or 7q deletion	2/65(3.0%)
5 or 5q deletion	2/65(3.0%)
Adverse karyotype	10/65(15.30%)
Complex,≥3 clonal abnormalities	6/65(9.20%)
HMA history, no. (%)	
Yes	10(12.70%)
No	69(87.30%)
MDS history, no. (%)	
Yes	3(3.80%)
No	76(96.20%)
Cycle of therapy, median (min, max)	6(4–12)
Gene mutations	
ASXL1	10(12.6%)
BCOR	7(8.8%)
CEBPA	11(13.9%)
EZH2	6(7.5%)
FLT3	14(17.7%)
KRAS	1(1.2%)
NPM1	14(17.7%)
NRAS	12(15.1%)
RUNX1	4(5.0%)
SRSF2	2(2.5%)
TP53	5(6.3%)
U2AF1	2(2.5%)
ZRSR2	3(3.7%)
STAG2	5(6.3%)
SF3B1	1(1.2%)
BCORL1	4(5.0%)
DNMT3A	21(26.5%)
TET2	11(13.9%)
IDH1	8(10.1%)
IDH2	13(16.4%)
GATA2	4(5.0%)
KIT	3(3.7%)

ELN European Leukemia Net, HMA hypomethylating agents, ECOG PS Eastern Cooperative Oncology Group Performance Status.

Data presentation: Categorical, n (%); continuous, median (IQR).

### 3.2 Efficacy

**Figure 1:**
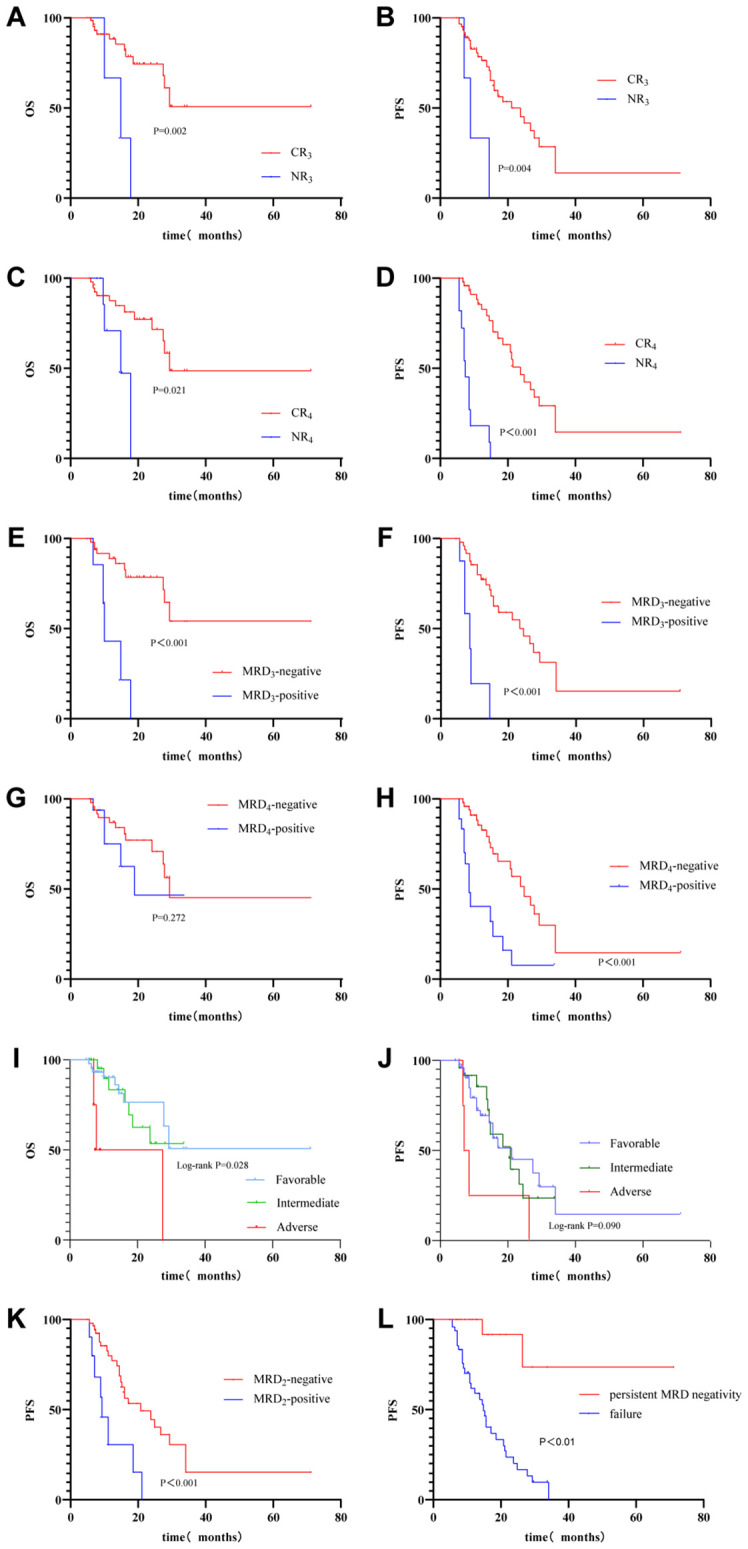
Overall survival and progression-free survival of unfit AML patients treated with long-course venetoclax and hypomethylating agents, stratified by treatment response, MRD status, and genetic risk category. **(A, C, E, G, I)** Overall survival (OS) curves for different response, MRD, genetic risk, and persistent MRD subgroups. **(B, D, F, H, J, K, L)** Progression-free survival (PFS) curves for different response, MRD, genetic risk, and persistent MRD subgroups. CRn, Composite complete remission achieved after n cycles of treatment; NRn, No remission achieved after n cycles of treatment; MRDn, Measurable residual disease status assessed after n cycles of treatment.

This study enrolled 79 patients, and treatment regimens along with efficacy assessments after each treatment cycle were systematically documented for all patients. The median overall survival (mOS) for the entire cohort was 11.5 months, and the median progression-free survival (mPFS) was 10.8 months (**Figure 1**). After one cycle, all patients with acute myeloid leukemia secondary to myelodysplastic syndromes (MDS/AML) and those with relapsed/refractory acute myeloid leukemia (R/R AML) achieved complete remission (CR). Among the evaluable population, the composite complete remission (cCR) rate after one cycle was 94.74% (72/76), and the rate of measurable residual disease negativity by flow cytometry (MRD^-^ rate) was 81.43% (57/70). After two cycles of treatment, two patients experienced disease relapse, including one patient with MDS/AML. Among the evaluable population, the cCR rate after two cycles was 95.89% (70/73), and the MRD^-^ rate was 85.71% (60/70). After three cycles, two additional patients experienced relapse, including one patient with R/R AML and one patient with an adverse-risk karyotype. Among the evaluable population, the cCR rate was 95.59% (65/68), and the MRD^-^ rate was 86.15% (56/65). After four cycles, a total of 11 patients had relapsed. Among the evaluable population, the cCR rate decreased to 83.58% (56/67), and the MRD^-^ rate was 75.00% (54/72). Within the four-cycle treatment period, 58.23% (46/79) of patients achieved sustained complete remission, and 46.84% (37/79) maintained a sustained MRD-negative status. Subgroup analysis revealed that 66.67% (2/3) of patients with MDS/AML and 100% (2/2) of patients with R/R AML ultimately experienced disease relapse.

### 3.3 Adverse reactions

In the enrolled patient population, the incidence of hematologic adverse reactions exceeded 50%. Adverse events greater than grade 3 were most commonly leukopenia (89.8%), anemia (78.4%), and thrombocytopenia (78.4%), all occurring at relatively high rates. Non-hematologic adverse reactions also occurred with considerable frequency. Grade ≥3 non-hematologic adverse reactions primarily included: concurrent fever/pneumonia (88.6%), electrolyte imbalances (25.3%), gastrointestinal adverse reactions (20.2%), liver function abnormalities (15.1%), coagulation abnormalities (11.3%), bacteremia (8.9%), and skin infections (6.3%).

### 3.4 Subgroup analysis by FAB subtype

**Figure 2:**
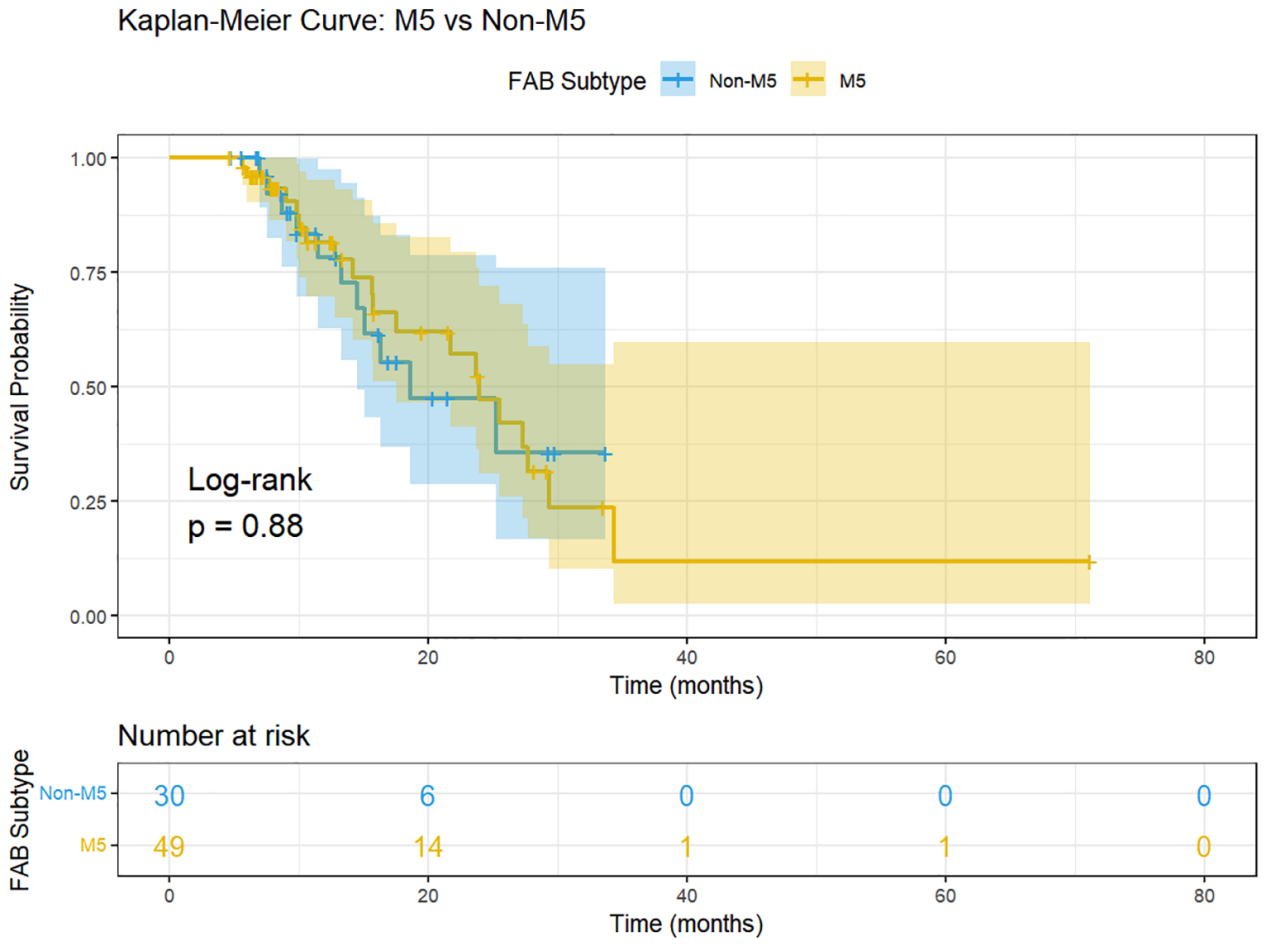
Kaplan-Meier survival curves comparing overall survival between M5 (n=49) and non-M5 (n=30) AML patients treated with long-course VEN-HMA therapy.

Given the high proportion of FAB M5 subtype (62.0%) in our cohort, we performed a subgroup analysis to evaluate whether FAB subtype influenced treatment outcomes. As shown in **Table 3**, no statistically significant differences were observed between M5 and non-M5 patients in CR rates (59.2% vs. 63.3%, P = 0.814), MRD-negativity rates (40.8% vs. 50.0%, P = 0.488), or median overall survival (**Figure 2**) (23.9 vs. 18.6 months, log-rank P = 0.878). Cox regression analysis confirmed that FAB M5 was not associated with inferior survival (HR = 0.94, 95% CI: 0.45–1.99, P = 0.878). Among patients who achieved CR, the MRD-negativity rate was also comparable between M5 and non-M5 patients (69.0% vs. 78.9%, P = 0.522). These findings suggest that the efficacy of long-course VEN-HMA therapy and the prognostic value of MRD monitoring are consistent across FAB subtypes.

**Table 3:** Comparison of outcomes between M5 and Non-M5 subgroups.

Outcome	Non-M5 (n=30)	M5 (n=49)	P value
CR rate, n (%)	19 (63.3)	29 (59.2)	0.814
MRD-negative rate, n (%)	15 (50.0)	20 (40.8)	0.488
Median OS, months (95% CI)	18.6 (12.3–24.9)	23.9 (16.8–31.0)	0.878
HR for OS (M5 vs Non-M5)	Reference	0.94 (0.45–1.99)	0.878
MRD-negative rate in CR patients, n (%)	15/19 (78.9)	20/29 (69.0)	0.522

Data are presented as n (%) or median (95% confidence interval). P values were calculated using Fisher’s exact test for categorical variables and log-rank test for survival comparisons. HR, hazard ratio; OS, overall survival; CR, complete remission; MRD, measurable residual disease.

## 4 Discussion

This single-center retrospective analysis investigated the efficacy of long-course (≥4 cycles) Venetoclax-based therapy and the core role of measurable residual disease (MRD) in patients with acute myeloid leukemia (AML) who are unfit for intensive chemotherapy due to age, performance status, or comorbidities. A total of 79 patients were enrolled. The composite complete remission (cCR) rate was 60.7%, with median overall survival (OS) and progression-free survival (PFS) of 11.5 months and 10.8 months, respectively. Despite the inclusion of 3 patients with secondary AML from myelodysplastic syndromes (MDS) and 2 with relapsed/refractory (R/R) AML, the overall outcomes were comparable to the cCR rate (66.4%) and median OS (14.7 months) reported in the pivotal VIALE-A phase III trial (6). The key finding of this study is that dynamic MRD monitoring provides critical prognostic information: patients who achieved sustained MRD negativity had favorable outcomes, while MRD reversion preceded clinical relapse. These results highlight the potential utility of MRD as a guide for treatment decisions in this patient population.

Dynamic MRD changes correlate with clinical outcomes. In our cohort, the MRD-negative rate increased from 81.43% after cycle 1 to 85.71% after cycle 2, but declined to 75.00% after cycle 4, coinciding with a cumulative relapse count of 11 patients. This pattern suggests that while early MRD negativity is a strong indicator of favorable response, a single timepoint MRD-negative status does not guarantee long-term disease control. Some patients may experience MRD reversion to positivity and subsequent relapse during the disease course. Notably, patients with sustained MRD negativity for at least 4 cycles (46.84% of the cohort) demonstrated significantly superior progression-free survival compared to those with persistent MRD positivity or fluctuating MRD status (P < 0.01), confirming MRD as a critical prognostic biomarker in AML patients receiving VEN-HMA (9). These findings underscore the necessity of continuous, dynamic MRD monitoring rather than reliance on single timepoint assessments.

The prognostic value of MRD monitoring has been demonstrated across different treatment modalities. For instance, in the RELAZA2 trial, which evaluated azacitidine as preemptive MRD-directed therapy in patients who had achieved remission after intensive chemotherapy or allogeneic transplantation, 63% (60 patients; 95% CI, 54-71%; P<0.0001) of MRD-positive patients treated with azacitidine remained relapse-free at six months. In patients who achieved sustained MRD-negativity, 60-month OS and relapse-free survival rates were 88% and 79%, respectively, highlighting the excellent prognosis associated with persistent MRD negativity (10). While the treatment context differs from our study of continuous VEN-HMA in unfit patients, these findings collectively underscore the clinical significance of MRD monitoring in AML.

Furthermore, subgroup analysis based on the ELN 2024 genetic risk classification for low-intensity therapy revealed that final CR and MRD-negative rates were 68% and 42% in the favorable-risk group, both 54.17% in the intermediate-risk group, and only 20% in the adverse-risk group. The low MRD-negative rate in adverse-risk patients aligns with their high risk of relapse, suggesting that VEN-HMA alone is often insufficient for achieving deep MRD clearance in this population. For these patients, treatment strategies must be dynamically adjusted based on MRD monitoring.

For patients with persistent MRD negativity, long-course VEN-HMA effectively controls disease progression without requiring treatment intensification, balancing efficacy with reduced toxicity. Conversely, for patients with persistent MRD positivity or reversion after negativity, timely intervention is critical to improve prognosis. Such patients likely harbor leukemic cells resistant to VEN-HMA or undergoing clonal evolution; continuation of the same regimen may lead to progression. Early treatment modification should be considered, such as incorporating targeted agents (e.g., FLT3 or IDH inhibitors) to create a triple combination, or bridging to allogeneic hematopoietic stem cell transplantation (allo-HSCT) upon disease control (11). In our study, among the 14 patients with FLT3 mutations in this cohort, 5 (35.7%) received a triplet regimen combining VEN-HMA with a FLT3 inhibitor (sorafenib or gilteritinib). Of these, 4 patients received the triplet regimen as first-line therapy, all of whom achieved complete remission (CR) and MRD negativity. The remaining patient, who did not respond to VEN-HMA alone after one cycle, achieved CR upon addition of gilteritinib. Prospective studies are warranted to validate the efficacy and safety of such approaches. The proposed mechanism involves synergistic multi-target inhibition, enhancing leukemic cell kill and improving MRD-negative rates, consistent with recent studies demonstrating high MRD-negative composite remission rates with targeted triple combinations in AML patients with FLT3 or IDH1/2 mutations (12, 13). Additionally, patients with MDS-secondary AML and R/R AML exhibited final relapse rates of 66.7% and 100%, respectively, with more pronounced MRD fluctuations, warranting increased MRD monitoring frequency.

Aligned with current advances in AML therapy, MRD-guided strategies are central to optimizing low-intensity regimens (14, 15). Our findings indicate that an efficacy assessment system centered on dynamic MRD changes enables precise treatment optimization. For favorable/intermediate-risk patients with sustained MRD negativity, treatment intervals might be adjusted based on performance status and toxicity to balance efficacy and safety. For MRD-positive patients, extending treatment cycles, intensifying the regimen, or switching therapies is necessary. MRD reversion predicts impending relapse more accurately than baseline molecular genetic markers currently used to guide transplantation decisions (16).

Integrating multi-omics technologies (genomics, transcriptomics) to analyze molecular profiles of MRD-positive patients could identify novel therapeutic targets, advancing personalized treatment strategies (15).

This study has several limitations that should be addressed in future research. First, its single-center, retrospective design introduces potential selection bias. The high proportion (62%) of FAB M5 subtype in our cohort reflects the referral patterns at our institution. Importantly, subgroup analysis revealed no statistically significant differences between M5 and non-M5 patients in CR rates, MRD-negativity rates, or overall survival (all P > 0.05), suggesting that the efficacy of long-course VEN-HMA therapy and the prognostic value of MRD monitoring are consistent across FAB subtypes. This mitigates concerns that the high M5 proportion may confound our primary conclusions. Nevertheless, validation in larger, multi-center cohorts with broader FAB subtype representation is warranted. Multi-center, prospective cohort studies are needed for validation. Second, the relatively small sample size (n=79) limits statistical power, precluding in-depth analysis of treatment response and MRD dynamics across different genetic mutation subgroups, and hindering definitive assessment of optimal treatment duration and MRD monitoring frequency. Third, the absence of a control group precludes direct comparison of efficacy between long-course and short-course VEN-HMA, and makes it difficult to definitively determine the superiority of MRD-guided therapy over conventional fixed-duration treatment. Randomized controlled trials are required to provide higher-level evidence.

Survivorship bias and interpretation of treatment duration. An additional methodological consideration is the absence of a control group of patients receiving short-course VEN-HMA therapy. As a result, the observed survival outcomes in patients who received extended treatment cycles (≥4 cycles) are subject to survivorship bias: patients who lived longer were inherently more likely to receive additional treatment cycles, regardless of treatment efficacy. Therefore, the association between longer treatment duration and favorable outcomes should not be interpreted as causal evidence that extended therapy directly improves survival. Rather, our findings suggest that patients who achieve sustained MRD negativity and tolerate treatment well are more likely to continue therapy and experience better outcomes. This distinction is critical for interpreting our results and highlights the need for prospective randomized trials with predefined treatment duration protocols to determine the optimal treatment course and to evaluate whether MRD-guided therapy offers advantages over conventional fixed-duration regimens.

## 5 Conclusion

In conclusion, dynamic MRD monitoring provides valuable prognostic information in unfit AML patients receiving VEN-HMA therapy. Patients achieving sustained MRD negativity have favorable outcomes, while MRD reversion precedes clinical relapse. These findings support the use of MRD as a guide for treatment decisions: continued VEN-HMA therapy appears appropriate for patients with sustained MRD negativity, whereas those with persistent MRD positivity or reversion may benefit from treatment intensification, including addition of targeted agents or consideration of allogeneic transplantation.

While extended treatment courses (≥4 cycles) were administered to all patients in this cohort, causal conclusions regarding treatment duration cannot be drawn due to the absence of a control group and potential survivorship bias. Rather, our findings suggest that patients who achieve sustained MRD negativity and tolerate treatment well are more likely to continue therapy and experience better outcomes. Prospective studies with predefined treatment duration protocols are warranted to validate MRD-guided strategies in this population.

## Data Availability

The original contributions presented in the study are included in the article/supplementary material, further inquiries can be directed to the corresponding author/s.
